# Workforce Readiness Training: A Comprehensive Training Model That Equips Community Health Workers to Work at the Top of Their Practice and Profession

**DOI:** 10.3389/fpubh.2021.673208

**Published:** 2021-06-08

**Authors:** Lily K. Lee, Elizabeth Ruano, Pamela Fernández, Silvia Ortega, Carlos Lucas, Maud Joachim-Célestin

**Affiliations:** ^1^Community Health Workers/Promotores Academy, San Manuel Gateway College, Loma Linda University, San Bernardino, CA, United States; ^2^School of Behavioral Health, Loma Linda University, Loma Linda, CA, United States; ^3^DAP Health, Palm Springs, CA, United States; ^4^School of Medicine, Loma Linda University, Loma Linda, CA, United States

**Keywords:** training, competency, skills, workforce readiness, capacity building, organizational readiness, model

## Abstract

**Background:** Recent reports have recognized that only 20 percent of health outcomes are attributed to clinical care. Environmental conditions, behaviors, and social determinants of health account for 80 percent of overall health outcomes. With shortages of clinical providers stressing an already burdened healthcare system, Community Health Workers (CHWs) can bridge healthcare gaps by addressing these nonmedical factors influencing health. This paper details *how* a comprehensive training model equips CHWs for workforce readiness so they can perform at the top of their practice and profession and deliver well-coordinated client/patient-centered care.

**Methods:** Literature reviews and studies revealed that training CHWs alone is not sufficient for successful workforce readiness, rather CHW integration within the workforce is needed. Consequently, this comprehensive training model is developed for CHWs with varying skill levels and work settings, and supervisors to support organizational readiness and CHW integration efforts. A systematic training program development approach along with detailed implementation methods are presented. Continuing education sessions to support CHW practice and Organizational Readiness Training for supervisors, leadership and team members directly engaged with CHWs in the workplace are also discussed. CHWs were involved in all phases of the research, development, implementation, and actively serve in evaluations and curriculum review committees.

**Results:** Components of the comprehensive training model are presented with an emphasis on the core CHW training. Two CHW training tracks are offered using three delivery modalities. Process measures with student learning objectives, outcome measures developed using the Kirkpatrick model to capture attitude, perceptions, knowledge acquisition, confidence, behavior, and overall experience, and impact stories by two CHWs are presented. Lessons learned from the implementation of the training program are discussed in three categories: Practice-driven curricula, student-centered training implementation, and adaptations in response to COVID-19 pandemic.

**Conclusion:** This comprehensive training model recognizes that training CHWs in a robust training program is key as the demand for well-rounded CHWs increases. Furthermore, a comprehensive training program must include training for supervisors, leadership, and team members working directly with CHWs. Such efforts strengthen the CHW practice and profession to support the delivery of well-coordinated and holistic client/patient-centered care.

## Introduction

Recent reports have recognized that environmental conditions, behaviors, and social determinants of health account for 80 percent of overall health outcomes while 20 percent are attributed to clinical care (1). The demands from these nonmedical factors have put a strain to an already burdened healthcare system calling for innovative approaches. In California, value-based care approaches have received increased support with improved health care processes and outcomes that led to expanded access for the uninsured. These efforts have made way for non-traditional services for patients with complex health and social needs (2). More work is ahead, nonetheless, as public health care systems focus on vulnerable populations such as those who experience homelessness, behavioral health issues, and other conditions stemming from the social determinants of health. Community Health Workers (CHWs)—and these include promotores, community health representatives, and individuals with many other titles delivering CHW scope of work—are ideal for addressing the needs of the high-risk, high-touch patient populations by increasing patient knowledge, activation, and adherence, while making an impact on patient health, health equity, and health care savings (3). Because CHWs often share ethnicity, socio-economic status, health conditions, and other barriers as well as assets with marginalized communities, CHWs have a unique understanding of their community. CHWs' ability to communicate and connect with people in a compassionate, caring, and culturally sensitive manner, and their expertise in health and social systems navigation make them ideal candidates to help individuals, families, and communities take full advantage of system resources. Particularly for patients with complex chronic conditions who need more intensive services, CHWs can serve as critical members of the healthcare delivery team and facilitate physical and behavioral health services along with community services and resource support (4).

Despite decades of studies on CHW efficacy and workforce development, and even classification in the U.S. Bureau of Labor, there are no national standards, curricula, or educational/career paths for preparing CHWs for workforce integration. Growing interest in CHWs and implementation of CHW programs are also drawing more attention to the need to train CHW supervisors and health system leaders who are unfamiliar with CHW selection criteria, training, scope of practice, roles and responsibilities, workload, reimbursement, and outcome measures (5, 6). A recent policy statement by the National Association of Community Health Workers (NACHW) further describes the value in building capacities of CHWs and the members of their team to achieve equity and social justice:

“*When the roles and leadership capabilities of community health workers are actualized and their organizing infrastructure is cultivated, CHWs can join with other professions to co-create and implement programs, practices, and policies that achieve health, racial equity, and social justice.”* (7).

In a statewide stakeholder engaged effort, the California Health Workforce Alliance (8) recognized the importance of establishing: “a competency-based framework” for CHW training that clarifies the *full range* of competencies, skill development for working in community-based primary and preventive care, and CHWs as a link between public health and health care. A more recent work further validated a set of measurable competencies linking to a workforce framework as a model for advancing the CHW profession (9). The California Health Care Foundation also continues to convene CHWs and CHW allies to further discuss the future of CHWs in the state (10, 11).

Currently in California, there is no legislation for CHW certification or credentialing (5, 12). As a result, there are a wide array of trainings to meet the needs of the CHWs scope of work. These trainings generally fall along the spectrum of grassroots/community trainings, in-house/on the job trainings, issue or topic specific trainings, and structured/more academic trainings (some granting college credits). The training described in this paper shows a model that utilizes a comprehensive set of competencies to strengthen CHW workforce readiness. The training helps to actualize CHW potentials to deliver a wide array and full range of roles and responsibilities as health care team members. The model also includes continuing education (CE) for CHWs, and Organizational Readiness Trainings (ORTs) for supervisors, team members, and leadership to maximize CHW potentials and integration efforts. The comprehensive training program was designed and has been implemented since 2016 by the Community Health Workers/Promotores Academy (hereinafter the Academy) at San Manuel Gateway College, Loma Linda University, in California.

Since 2016, the Academy has trained 11 cohorts under this training model. Diverse cohorts included mostly female adult students (8:2 female to male ratio) with high school degrees or GED with no experience to 20 plus years of experience from all ethnic and racial backgrounds (predominantly Hispanic, then African American, Asian, and Native American). Students who successfully complete the training program receive a certificate from Loma Linda University. Graduates have been hired in over 40 different organizations (community, school district and mostly health care settings) across three counties (Los Angeles, Riverside, and San Bernardino).

## Pedagogical Framework, Principles, and Standards

The Academy's training model strengthens the CHW capacities for workforce readiness by offering a comprehensive breadth of competencies (knowledge, skills, abilities) to perform various roles and to deliver a broad scope of services alongside clinical care teams in various complex service delivery settings. The training model promotes workforce readiness capacities so graduates can perform at the top of their practice and profession regardless of the workplace setting.

**Figure 1:**
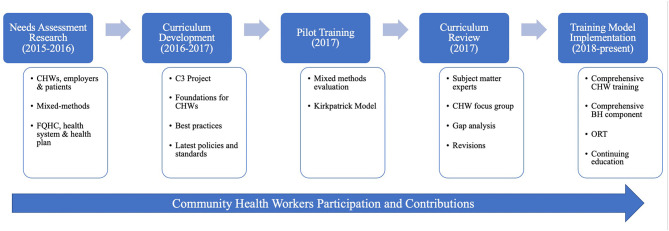
Comprehensive training model development.

Training content and materials were developed and informed by a systematic approach, including: findings from triangulated stakeholder-engaged mixed methods training needs assessment research (6, 13); curriculum development referencing work published by CHW experts, best practice guidelines, and policies; pilot testing and evaluation using mixed methods approach; curriculum review with subject matter experts to perform gap analyses; then the implementation of the comprehensive training model (see Figure 1).

All trainings are delivered with a practice-driven approach to strengthen the CHW workforce. Competency-based curricula are delivered using popular education approaches and adult learning andragogy that encourage adult students to actively contribute to their learning with their “shared lived” personal and work experiences. Special attention is given to application of *CHW-engaged strategies* through case-based scenarios, which have been developed to promote higher level critical thinking for real-life application and practice.

**Figure 2:**
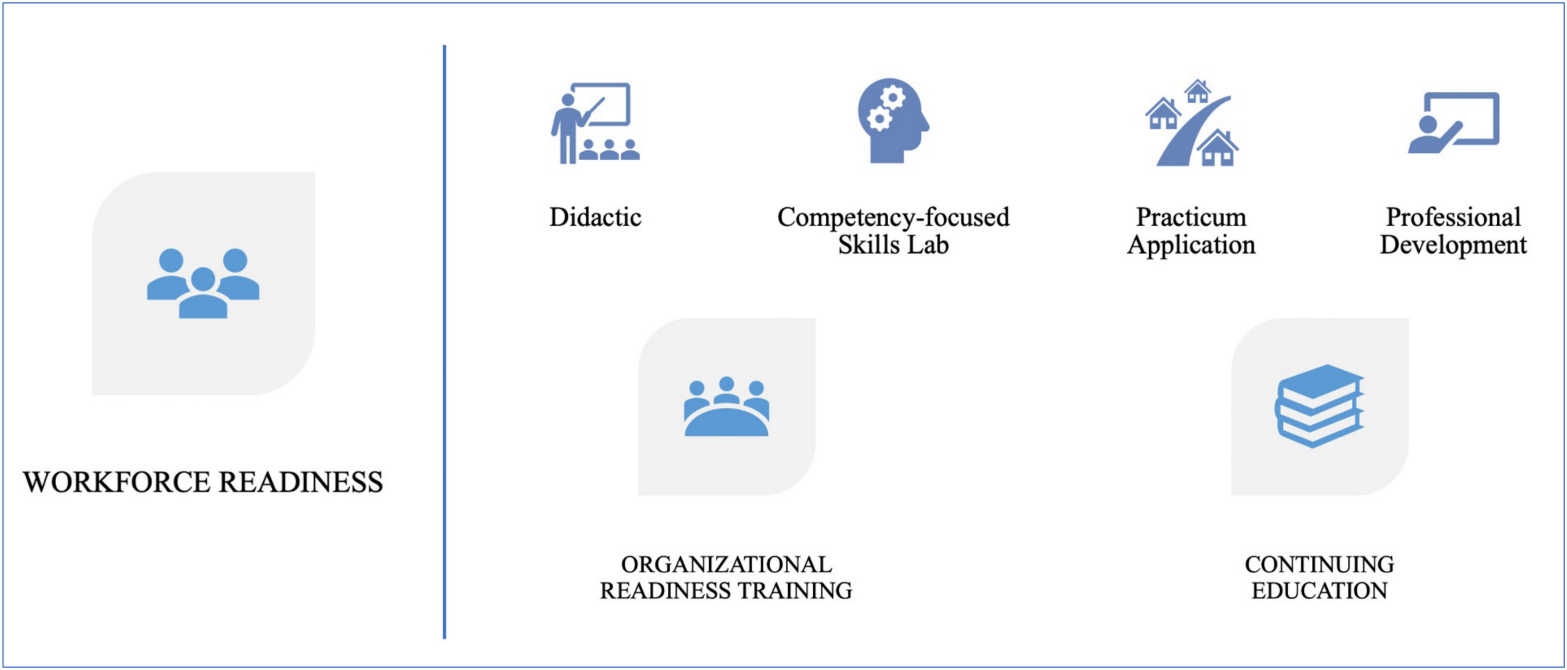
CHW Workforce readiness framework: A comprehensive training program.

The comprehensive training program includes: *Core CHW trainings* (offered in two different tracks through three different delivery modalities, in two languages, and training hours ranging from 200 to 400 hours), *Continuing Education (CE)* for CHWs, and *Organizational Readiness Trainings (ORTs)* for supervisors, leadership, and teams (see Figure 2).

The *core CHW trainings* contain four pillars to support workforce readiness: didactic instruction, skills lab with competency assessments, practicum application, and professional development capacity building. *Continuing education* and other specialty trainings are designed to support competencies needed for more specific areas of focus. Case-based learning and applications are central to the CE sessions. *Organizational Readiness Trainings* are unique to this training model as the need for a more systematic training for supervisors and leadership was identified through the needs assessment research (6, 13). A series of ORT sessions are intended for supervisors but are also offered to leadership and members of the team directly working with CHWs. The ORTs are customized to meet the needs of the organization, whether new or experienced, in delivering CHW-engaged programs as well as supporting the organizational training needs to prepare supervisors for their roles, to understand the wide array of CHW duties and responsibilities, addressing role clarity and communication among health care team members working with CHWs, and offering opportunities for supervisors across organizations to share lessons learned and best practices.

Competency-based curricula are updated and adapted regularly based on a rigorous evaluation design, stakeholder feedback, curriculum reviews, and gap analyses (see Figure 1 and Tables 1, 2). These processes involve CHWs from various settings as active contributors and are closely aligned with latest policies and guidelines to ensure the program meets best practice standards. The Academy's access to health sciences faculty at Loma Linda University offers an additional layer of internal expertise that is regularly called upon for curriculum developments, reviews, and training implementation.

**Table 1:** The academy's core competencies and main training topics.

**Community Health Workers/Promotores Academy Core Competencies:**		
Communication skills: Listening, reading, speaking, and writing		
Interpersonal skills: Teamwork, leadership, inter-professionalism		
Problem solving		
Critical thinking		
Professionalism and workforce capacity building		
**Foundations of CHW Practice**	**CHW and Behavioral Health**	**Clinic-based CHW Practice**
• Individual and community capacity building • Behavior change • Access to preventive health care • Direct services • Culturally humility and mediation • Health education, coaching and promotion • Informal counseling and social support • Advocacy and outreach	• CHWs and behavioral health basics • Principles of behavioral health practice • Prevalent mental health disorders • Skills for behavioral health care and service delivery • Cultural considerations in behavioral health care and service delivery • Risk assessment tools • Community Resiliency Model (CRM) • Mental Health First Aid (MHFA)	• Clinical-community networking principles and practice • Patient safety practices • Disease management practices • Care transitionmanagement • Clinical and community interventions and tools • Health coaching and support services

**Table 2:** Training program evaluation and Kirkpatrick model matrix.

**Evaluation**	**Indicators and Instruments**	**Implementation**	**Kirkpatrick model**
Process	Student Learning Outcome (SLO) Learning domain assessments	During training	Reaction and Learning
Outcome	Pre-post assessment Instructor evaluation survey Practicum evaluations survey End of program survey	Before and after training End of training End of training End of training	Learning and Behavior
Impact	Graduate survey and focus group Employer survey and interview	6–12 months post training	Behavior and Results

## Learning Environment and Pedagogical Format

For the purpose of this paper, the pedagogical format description will focus on the *comprehensive core CHW trainings*. CEs and ORTs utilize similar format and delivery methods. Discussions on the ORTs will be described in more detail in another paper.

**Training Tracks and Modalities**. The Academy offers traditional and intensive track training programs delivered in-person (in traditional classroom settings), hybrid (in-person and virtual) setting, or fully virtual/online. All training programs are available in English and Spanish. Hybrid and fully online training modalities use synchronous (live, real time engagement) and asynchronous (independent) instructions. Students have independent work to complete in Canvas (an online Learning Management System), as well as synchronous live online class times with the instructors and classmates via Zoom video conference application. Other software and online applications are utilized as applicable to facilitate instruction and communication. Under months of physical distancing regulations due to the Covid-19 pandemic, all training programs transitioned from in-person and hybrid to fully virtual. Because use of technology and computer skills are part of the core training to build professional development capacities, the transition to online learning was met with minimal barriers.

**Multidisciplinary Team**. Trainings are taught by a team of instructors with subject matter expertise from various disciplines, including experienced CHWs, masters and doctors in public health, licensed clinical social workers, marriage and family therapists, psychologists, nurses, and medical doctors. Additional experts and faculty in specific health issues (e.g., substance use), Community Resiliency Model (CRM) and Mental Health First Aid (MHFA) fields engage throughout the training program depending on needs and scope.

**Curricula Organization**. The training materials are organized in three main components: didactic instruction, competency-focused skills lab, and practicum application. Professional development is embedded in each of these three components. Units and modules contain student learning objectives based on the Blooms Taxonomy with the application of the three learning domains: cognitive (thinking), affective (emotions or feeling) and psychomotor (physical or kinesthetic) (14). Core Competencies and topics covered in the Clinic-based CHW Intensive Training Program are shown in Table 1.

### Didactic Instruction

Didactic instruction is delivered through interactive, participatory methods, using popular education principles to build upon students *shared lived* experiences and built expertise. Student learning assessments are embedded throughout as part of process evaluation to capture student learning progress. Table 1 presents the Academy's core competencies for the Clinic-based CHW intensive Training Program. The school-based and other specialty training programs are not included in this paper.

### Competency-Focused Skills Lab

Skills labs focus on competencies using case-based scenarios to bridge gaps between the didactic training and practice. These competencies focus on CHW-engaged strategies that straddle the health care system interventions and community-clinical links domains described in a recent report by the Centers for Disease Control and Prevention (3). Through case scenarios, CHWs work on demonstrating various skills necessary to implement *CHW-engaged strategies*, including motivational interviewing for behavior change planning and harm reduction, patient navigation through transitions of care, home visitation (tele/virtual visitation), medication review, accompaniment, and more.

The content design and teaching approaches for the skills lab training and competency testing are unique to the Academy's training model. As scholars agree, adult learning requires an intentional *learning transfer* approach that scaffolds around “meaningful social interaction and the development of transfer skills” (15). The Academy's competency-focused skills labs are built upon four premises that promote *learning transfer*: (1) Building up of a new CHW self-image; (2) Identifying and emphasizing critical gaps; (3) Building on prior knowledge; and (4) Chronological thinking.

*Building up of a new CHW self-image* is based on the premise that working as a CHW is not a job, it's a vocation. Instilling a pathway to support CHWs' passion, belonging, and a strong sense of identity is the first step in training a CHW to be successful (15). Within the framework of a popular education teaching environment, all are encouraged to view themselves as active contributors to the teaching rather than being recipients of knowledge taught by “experts” alone. Throughout the course, multiple opportunities are created for team-learning, where students collaborate in small group projects leveraging their strengths and extra-curricular skills and experiences as complimentary learning opportunities. The goal is to support students as they identify their endogenous and unique strengths that they may not have believed or perceived to exist within them and contribute to the betterment of the healthcare system.

*Identifying and emphasizing critical gaps* focuses on the role of CHWs in filling these gaps within the current healthcare system, and how this process helps CHWs recognize their position as contributors to the team and the organization. Students are guided through exercises that help identify gaps in the current healthcare system and provider services, such as the “great divides” in literacy, income, culture, and others that contribute to high healthcare cost. The training also emphasizes the process of identifying gaps in CHWs' own learning (16) by approaching each individual from a holistic perspective, aiming to ensure that every need particularly in the areas of social determinants of health is addressed with a patient/person-centered approach.

*Building on prior knowledge* is based on the premise that learning is more deliberately transferred when there is mindful abstraction of prior knowledge in new relevant contexts (16, 17). Because CHWs vary widely in personalities and skills as well as educational, experiential, socio-economic, cultural, and professional backgrounds, instructors encourage logical thinking to connect life experiences and previously acquired knowledge to conclusions (15). Thus, they learn by association. Other strategies and tools such as color-coding, easy-to-remember mnemonics, life stories and life principles, and pattern identification are used to further build knowledge connections. As CHWs develop more confidence and become more comfortable with their roles, CHWs can build upon these learning processes to apply to specific circumstances. Another part of the process of building connections includes helping students identify their learning style, providing them with opportunities to adapt the material to their own style and asking those with similar interests/learning styles to share the newly learned concepts with each other (18). This process of conceptualizing, creating logical ways to retain information and associating new content to previous knowledge serves to reduce the burden of learning, increase long-term retention, and allow students to make more informed decisions in the future.

*Chronological thinking* is based on the premise that practice crosses over different contexts and contents over time, improving adaptability and preparedness. As implied in the previous section, activities in this training are mostly described as combinations of strategies and tools which lead to the creation of several protocols, all of which are written within the frame of a timeline (before, during, and after). Once the didactic units are covered, CHWs are then asked to adapt appropriate strategies and tools to a variety of circumstances in the setting of skills labs. These skills labs place the knowledge acquired into real life scenarios in which students must think of practical ways to apply what they've learned in the context of their community or work setting. As much as is feasible, the skills lab environment is created to simulate reality and allows for role playing episodes and interactive activities with “patients/clients/members.” Competencies exam stretches the students' abilities to apply their newly acquired learned skills with unpredictable patients/clients. It also helps them interact better within the setting of a team. Offering multiple opportunities to apply their knowledge to real life applications is *reinforcing the learning transfer process* (15); it ensures that all students become more confident and better prepared to face the workforce demands.

### Practicum Application

Practicum is a vital portion of the core comprehensive CHW training program, giving students real-life opportunities to further develop, practice, and refine the application of competencies covered in the didactic and skills labs. Students are required to complete a series of assignments based on Logic Model principles (19) that lead to a final Community Engagement Project (CEP) or Community Diagnosis Project (CDP). Both the CEP and CDP provide students the opportunity to look deeper into the communities they serve, identifying current health or social issues. The research tools for these projects include windshield surveys, key informant interviews and/or focus groups, research on best practices and strategies, and findings from patients' success stories. With systematic tools and methods to survey communities, and their expertise, students have an opportunity to critically reflect and process potential solutions to create a practical and innovative program action plan. Depending on the program track and practicum settings, students often can implement the proposed program plan. The practicum experience concludes with an oral presentation where students highlight their findings reflecting community perspectives on social and/or health issues, spotlighting best practices, recommendations, and providing activities that involve *CHW-engaged strategies* to address issues/barriers shared by the community. Practicum projects and scope of work may vary by site, track, and training modalities; therefore, projects reflect unique approaches to specific assigned responsibilities and priorities of the practicum site. Students also have the opportunity to network with other health and social service professionals and form alliances with members of the community. Using an adult learner knowledge transfer perspective, this practicum application allows students to actively incorporate new knowledge and skills in a relevant context by telling the “story” from the community perspective. Scholars refer to this learning application as *organizational mirroring and owning the learning*, which is a “lever” of change that influences learning transfer (15, 16).

## Results, Processes, and Tools

Parallel to the development of the CHW training curricula was the creation of a robust evaluation plan to assess process and outcome measures of student learning and program effectiveness. Like the student learning assessments, programmatic outcomes are captured using evaluation instruments created based on the Kirkpatrick's Model (20). Program evaluation captured three measures on training methods, eight on “on-the-job application” of competencies, and one open-ended question on the positive effects or outcomes CHWs might have experienced as a result of applying what they learned back on the job. Although not within the scope of this paper, evaluation reports using the aforementioned measures have been produced and presented to training partners and stakeholders. To further capture training impact, graduate and employer evaluations are administered 6–12 months post training. In addition to evaluation tools, groups of subject matter and assessments experts, post-training CHW focus groups, and curricula review further guided and continue to guide improvement plans to ensure program and curriculum efficacy in strengthening the CHW workforce readiness capacities (see Table 2).

The Academy has trained 308 culturally diverse CHWs since its inception in 2012, of which 196 were trained under this robust and comprehensive training model: one pilot cohort (*N* = 14), seven cohorts (*N* = 158) in the intensive track Clinic-based CHW training and three cohorts (*N* = 24) in the traditional track Foundations and Behavioral Health training programs. End of program evaluations report 98% retention rates and 99% job placement rate within 6–9 months of the training, where 90% of students are employed in clinical, community and school-based settings. CHWs from the intensive clinic-based CHW training program were hired in 39 different healthcare organizations across three counties in Southern California; traditional track CHWs were hired by two Community-Based Organizations to work in local communities and school districts. During time of enrollment, about 10% of students reported having previously pursued higher education and attained degrees in social work or public health or were enrolled in graduate programs. Reported attrition was due to health issues and/or personal/family obligations. Pre-post assessments show an average 34% increase in knowledge acquisition, and instructor evaluations averaged 4.5 ratings in an eight-item Likert-scale, where 1 = poor and 5 = excellent. More than 85% of all CHWs agreed or strongly agreed that the competency trainings prepared them with knowledge, skills, and abilities to perform and deliver patient-centered care services. Through the open-ended responses, the following themes emerged that describe CHWs' learning experiences: CHWs felt better equipped with necessary tools for practice; CHWs expressed increased capacity to have more meaningful patient engagement and experience; and CHWs felt they are able to make better contributions to their health care organizations, community and patients. These sentiments are described directly by a few CHWs' open ended responses:

“You will truly learn a lot about yourself while learning how to give to others.”

“This training is invaluable.”

“Take advantage of your time at the Academy and apply your skills immediately. Put it into practice.”

## Discussion

### Lessons Learned

Designing, developing, and implementing a comprehensive training program requires commitment to serve and build equity in the community, a process that should involve the investment of strengthening the CHW workforce (a group of professionals ideal to make an impact). Over the course of 4 years, 11 cohorts, and series of rigorous evaluations, several lessons learned merit attention. The lessons learned are presented in three categories: Practice-driven curricula, student-centered training implementation, and adaptations in response to COVID-19 pandemic.

**Practice-Driven Curricula**. (1) The practicum projects provided real-life application within a real workplace environment. Students found research methods such as windshield surveys, key informant interviews, focus group highly valuable as it allowed them to see and understand in depth their communities' needs and existing resources. Creating a safe space and time for students to share their experiences and present highlights of their practicum are recommended; they promote reflections on their CHW practice, networking, professional growth, and comradery much needed among CHWs whether from similar or different geographical regions; (2) Competency-focused skills labs were highly rated by students. Logistics for competency exams should be organized with detailed time breakdown, rotation cycles, rubric, and supporting materials. Guides should be made for both the instructors and students, distributed in advance for transparency and preparation.

**Student-Centered Training Implementation**. (1) The comprehensive nature of the training offers large amount of content that may be challenging to organize and retain. Study guides, review sessions, instructors/staff availability for one-on-one office hours, and study skills building workshops helped supplement the learning gaps and support learning and retention; (2) The interdisciplinary team-teaching approach offered students opportunities to grow confidence and gain a clearer understanding of their role as they worked through case scenarios with other professionals during the training. Perspectives were gathered using mixed methods assessment of the value of a multidisciplinary training approach and presented at the 2019 American Public Health Association Annual Conference. One CHW describes this experience:

“*First of all, I had no experience working in a clinic setting. So, I felt very lost, even some of the terminology was a little scary. However, having all these clinicians helped me understand the different roles and services that CHW's can offer. I had no idea that as a CCHW* [clinic-based CHW] *we would be able to offer informal counseling, disease management or even mental health aid. Having all of our clinicians here was distinctly helpful.”* (CHW – Intensive Clinic-based CHW Training Program, Cohort 2)

(3) Teaching and promoting self-care and peer support throughout the training was deemed highly important and beneficial. Because CHWs share lived experiences, CHWs may experience vicarious trauma and be at higher risk of burnout. The behavioral health portion of the training not only provided CHWs with tools to assist others in areas of behavioral/mental health, but they were key fundamental skills that CHWs applied to their own mental health self-care practices.

**Adaptations in Response to COVID-19 Pandemic**. (1) Training materials and delivery methods required adaptations to facilitate online learning. A 2-week break at the onset of the “shelter in place” orders allowed CHWs and their organizations to make work accommodations, and for the Academy to transition to online platforms and conduct in-services to train staff on maximizing the use of technological applications; (2) Utilizing Canvas provided students with full access of all the materials. Zoom application and its features allowed for live, synchronous learning sessions with creative adaptations to activities to promote participation. The Remind application along with Canvas announcements directly delivering messages to email inboxes and phones facilitated continuous communications; (3) Evaluation and assessments were adapted to utilize Zoom features and Qualtrics. The number of assignments and assessments to measure student learning outcomes (SLOs) were adjusted to include shorter and more frequent quizzes to provide self-assessments and progress; (4) Participation in a fully online training requires devices and dependable internet. Thanks to gracious donors, the Academy was able to offer desktops and iPad loaners. Additional trainings on utilizing smartphones and other personal devices further supported the transition process.

### Practical Implications

Real life impact and practical implications of the comprehensive training program can be depicted by real life testimonies of graduates of the program. These testimonies represent the Academy's efforts to center CHWs experiences in the training and draw upon lessons learned to engage in active efforts for the betterment of the training. The following two CHW stories depict how “a typical CHW is [as] anything but typical” (5) and how this comprehensive training impacted their personal and professional lives.

**Silvia's Story**. It has been over a decade since I was called to do this work, overcoming personal health challenges and life altering events. When I was first introduced to community health profession, I quickly realized that I had *already* been engaging in these community and health-strengthening activities. I felt the need to formalize my calling and explore available trainings to serve my community in a more profound way. That is when I decided to attend the Community Health Workers/Promotores Academy Foundations of CHW Practice training.

The training solidified for me that I was born to do this work. The training was extremely valuable, specifically because it was the first time my lived experiences were drawn upon as sources of knowledge and guidance for others. I was trained to deliver and perform CHW competencies and skills in a culturally appropriate manner. I enjoyed the popular education activities and learning about the social ecological model. I was able to learn about potential health outcomes of those that live within the spectrum of the community framework. For example, drawing upon my own lived experiences and leveraging cultural humility, I was able to support pregnant women with their maternal health, although I had never been pregnant myself. My confidence flourished as a result of this training, and I found the courage to run and win a campaign for school board office. The training impacted my community actions as a school board leader who stood for change and advocated using public policy.

My training continued when I was given the opportunity to return to the Academy and participate in the first Community Health Worker clinic-based training. This training was profound personally, as my father was diagnosed with a life-threating disease during this same time. Instinctively, I became my father's CHW and experienced first-hand the need for comprehensive care. The clinical training allowed me to understand that physical health must be comprehensive and include emotional and spiritual health. Through the clinic-based training, I was able to acquire a valuable CHW lens and learn more about the root issues impacting community health. I saw the opportunity to learn more about behavioral health, and that is when I joined the committee to pioneer the behavioral health training at the Academy, incorporating the CRM model. The popular education approach highlighted the notion that individuals and communities can heal together and go from being trauma-informed to trauma-transformed. Now, after years of implementation, I serve on the behavioral health curriculum review committee where I work toward enhancing the training like I did before. The Academy has given me an incredible opportunity to come full circle as a student, graduate, and now instructor, where I can share with students what I have learned over the years.

**Carlos' Story**. Trust, by definition, implies that someone has confidence in you and believes in you, your character and strength. Working as a registrar, I was recognized for being compassionate, sincere, and resourceful. My ability to connect with diverse patients, building trust and comfort-level, was noticed by my supervisors. My responsibilities grew, and I was tasked with navigating and linking Transgender-identified individuals to care. This catapulted me in my upcoming career as a CHW. I was identified as someone who would be a perfect candidate to receive the training at the Academy at Loma Linda University San Manuel College and become a certified clinic-based Community Health Worker (CCHW). This opportunity has changed my career path completely. I quickly understood the need for this valuable role in this population and understood how my upbringing and intuitive sense of connection is valuable.

The Skills Lab training helped me prepare for situations like home visits and accompaniments. In one case, I was able to identify the cause of poor blood sugar management and coordinated the delivery of a new glucometer to the patient's home. After assessing the patient's living situation and eating habits, I provided education on cultural food choices and made recommendations to avoid dangerously low blood sugar levels. Working with patients in their home allowed me to recognize opportunities for improved mental health using informal counseling, demonstrating grounding and breathing techniques from the training. Learning about the CRM has allowed me to keep patients in a resilient zone and to identify the root causes of their stress. Motivational interviewing is another tool I utilize frequently to positively reinforce and celebrate small health improvements and commitment to thrive. I have learned through my training and experience that when you set SMART goals with your patients, they must be achievable and realistic to keep your patients on track and accountable.

During my time at the Academy, I had an opportunity to present on Health Disparities within the Transgender Community, which led to an expanded role to join the Diversity and Transgender Committees. After the training, I was given an opportunity of a lifetime as a trained CHW to influence and help various communities as a Linkage to Care Specialist, Transgender Navigator, Early Intervention Specialist and Housing Case Manager. I feel accomplished and blessed.

The ability to connect with people and being trusted is a powerful tool. Being a CHW has elevated that quality to all my job descriptions. The accomplishment came into a full circle when I was invited back to the Academy to speak on a panel about my experiences as a CHW. At that point I became a mentor and felt responsible to continue my journey into expanding my career as a CHW and participate in any possible CE training. I know this will lead me into an amazing future and am truly grateful that I am part of something grand.

## Conclusion

More than ever before, CHWs are being recognized as the critical workforce to address environmental conditions, behaviors and social determinants of health. Yet no standards have been established to assess and ensure quality training for CHWs nor to prepare organizations that employ them to integrate them into their teams. The Academy's comprehensive training model addresses both. The comprehensive core CHW training programs enable CHWs to perform at the top of their practice and profession in a variety of settings, promoting high standards of professionalism, a culture of critical thinking, as well as learning, innovation, and creativity. Just as importantly, the ORTs provide organizations hiring and overseeing CHWs with the opportunity to create a nurturing environment that facilitates CHW integration and long-term success. The key is not *who* trains the CHWs, but *how* they are trained and equipped for workforce readiness and for scaling-up the workforce to narrow the gaps in our nation's healthcare system.

## Data Availability Statement

The data supporting this article can be made available by the authors without reservation.

## Ethics Statement

Ethical review and approval was not required on human participants in accordance with the local legislation and institutional requirements. The participants provided their written informed consent to participate in quality improvement evaluations of the program. Written informed consent was obtained from individual(s) for the publication of identifiable data included in this article.

## Author Contributions

LL conceived and developed the framework. ER facilitated data analysis. ER, PF, and MJ-C contributed to the design of the implementation of the trainings. LL led the writing process. All authors contributed equally to the writing and editing.

## Conflict of Interest

LL, ER, PF, MJ-C, and SO declare employment at Loma Linda University during the time the work was submitted. CL and SO are graduates of the CHW/P Academy.
